# Translation and validation of the WHO-5 General well-being index into native language Quechua of the Peruvian South

**DOI:** 10.1016/j.heliyon.2023.e16843

**Published:** 2023-06-01

**Authors:** Renzo Felipe Carranza Esteban, Oscar Mamani-Benito, Julio Cjuno, Madona Tito-Betancur, Susana K. Lingán-Huamán, Dennis Arias-Chávez

**Affiliations:** a Grupo de Investigación Avances en Investigación Psicológica, Facultad de Ciencias de la Salud, Universidad San Ignacio de Loyola, Lima, Peru; b Universidad Señor de Sipán, Chiclayo, Peru; c Universidad Privada Norbert Wiener, Lima, Peru; d Universidad tecnológica del Perú, Arequipa, Peru; e Universidad Continental, Arequipa, Peru

**Keywords:** Translation, Validation, General well-being, Quechua, Peru

## Abstract

To translate and validate the WHO-5 General Well-being Index for the people of the Peruvian South, a cross-sectional instrumental study was carried out with the voluntary participation of 186 people of both sexes between the ages of 18 and 65 years (M = 29.67 years old; SD = 10.94) living in the south region of Peru. The validity evidence was assessed based on the content using Aiken's coefficient V according to the internal structure through a confirmatory factor analysis, and reliability was calculated through Cronbach's alpha coefficient. The expert judgment was favorable for all items (V > 0.70). The unidimensional structure of the scale was confirmed (χ2 = 10.86, df = 5, p = 0.05; RMR = 0.020; GFI = 0.980; CFI = 0.990; TLI = 0.980 y RMSEA = 0.080), and it presents a suitable reliability range (α = > 0.75). This shows that the WHO-5 General Well-being Index for the people of the Peruvian South is a valid and reliable scale.

## 1 Introduction

One topic of interest within positive psychology is the well-being study [1]. Historically, researchers have recognized the existence of two classical currents for its interpretation: the hedonistic tradition, focused on the subjective experience of pleasure or “feeling good” (subjective well-being), and the eudaimonic tradition, focused on the personal growth and realization of the human being (psychological well-being) [2].

Subjective well-being is defined as the degree to which a person feels that their life, generally speaking, is good. This perception results from a subjective self-appraisal of the quality of life [3]. In recent years, there has been a significant increase in research on subjective well-being, which has resulted in major contributions to areas such as health and healthy behaviors [4], organizational behavior [5], teaching–learning processes [6], and mental health in times of the sanitary emergency caused by the COVID-19 pandemic [7].

Despite this empirical evidence, studying well-being in indigenous communities is still a challenge, mainly due to language barriers because it has always been difficult to carry out studies when it is necessary to communicate speaking native languages [8]. This was evidenced in a study conducted with the Inuit people in Canada, where difficulties arose in measuring well-being and mental health aspects. It was even more difficult with another group called the Métis, where it was nearly impossible to gather information [9].

Faced with these limitations, it is likely that indigenous peoples are among the most vulnerable groups due to the adverse impacts of mental health issues, as it is not easy to develop prevention and health promotion strategies without accounting for their idiosyncrasy and language [10]. That was the case with the Kichwa people from the Ecuadorian Altiplano, who, due to their ancestral culture and tradition, were reluctant to receiving sexual education to prevent the spread of HIV [11]. Taking these events into consideration, some countries are implementing promising cross-sectional interventions. For example, in Australia, United States, Canada, and New Zealand, where the integration of services include intervention through community workers that speak the native language of the people to be intervened [12].

Peru is no stranger to this problem like many other countries of the region where native languages are dominant. The importance of studying mental health accounting for ideological, traditional, and ancestral aspects is highlighted. For example, a study that analyzed the approach to mental health for Colombian indigenous peoples [13] found that they do not understand the concept of mental health but view it from a holistic ancestral perspective, where the focus is on indicators such as good living, spirituality, harmony with mother earth, etc. Furthermore, another study carried out with Ayunara Chilean women sought to understand the meaning of Suma Qamaña in the Aymara language [14]. This phrase expresses the good way of living or to live fully (an element that can be found in the subjective well-being), and its interpretation is based on the relationships that are established with “others” (their community), which greatly differs from the interpretation in Spanish.

One of Peru's main characteristics is that it is a multicultural country. From time immemorial, it has been occupied by different peoples with their diverse customs and ways to understand life and well-being [33]. One of these peoples communicates using the native language Quechua, with Amazoni, Northern, Central, and Southern variants. The last one is divided into two subvariants: Quechua Chanka and Quechua Collao, used in the districts of Apurímac, Cusco, Puno, Arequipa, and Moquegua [15].

Faced with the need to intervene in rural areas where most indigenous people that speak native languages live and taking into account that they tend to be the most affected by armed [16] and public health [[17], [34]]; conflicts, there arises the need to count on culturally-adapted instruments to assess subjective well-being. This led research to consider instruments that have been proved to have appropriate psychometric properties in different countries of the region. Thus, the WHO-5 General Well-being Index stands out, which originated in 1982 when Europe required a measure to identify depression cases [18].

Today, there are short versions (WHO-5) that have been proved to be valid and reliable in countries like Peru [19], Colombia [20], and Argentina [21], thereby becoming a good alternative due to its psychometric performance and ease of application as a 5-item scale. Therefore, this study aimed to translate and validate the WHO-5 General Well-being Index into the Collao variant of the Quechua native language spoken in Puno, Peru.

### 1.1 Goal

To translate and validate the WHO-5 General Well-being Index for the people of the Peruvian South.

## 2 Method

### 2.1 Design

This is a cross-sectional instrumental study [22] for the adaptation and validation of the WHO-5 General Well-being Index for the Quechua-speaking population of Puno, Peru. Puno is a Peruvian High Andean city with about 1,172,697 inhabitants, of which 464,231 inhabitants reported to be Collao-variant Quechua speakers. They live in the northeast of Puno, mainly in the provinces of San Román, Azángaro, and Melgar. Their main economic activities are agriculture, livestock, and mining (National Institute of Statistics and Computer Science [ [23].

### 2.2 Participants

The non-probability convenience sampling method was used to determine the sample, applying inclusion criteria, such as communicating in Quechua (Reads, Writes, and Understands), being of legal age, and accepting informed consent. In addition, the exclusion criteria were to understand Quechua only but not to speak and write. Although not included in the demographic questionnaire, participants were recruited in areas where economic activities related to street commerce, agriculture and livestock, and interprovincial transportation are developed, and it resulted in a sample of 186 Quechua speakers, with a mean age of 29.67 ± 10.94. Regarding sex, it resulted in a proportional sample of 94 women (50.5%) and 92 men (49.5%). Most of them, 107 (57.5%), reported being single (Table 1).

**Table 1:** Characteristics of Puno's Collao-variant Quechua speakers.

	M	SD
Age	29.67	±10.94
	N	%
Sex		
Male	92	49,5
Female	94	50,5
Marital status		
Domestic partner		
Married	21	11,3
Single	45	24,2
Divorced	107	57,5

M = Mean; SD = Standard deviation.

### 2.3 Procedure

This research was developed in two stages: validation and translation of the WHO-5 General Well-being Index from Peruvian Spanish to the Collao variant of Quechua spoken in Puno, Peru. An authorization from the author of the original instrument was obtained by e-mail. The WHO-5 items were translated into Quechua considering the version previously adapted and validated for Peruvian Spanish by Ref. [19].

Before using the pilot text, the translation process was carried out following the recommendations of [22]; with an initial translation from a group of professionals, back translation by other translators that had not read the original questionnaire, a proofreading committee, and a pre-testing focus group.

#### 2.3.1 First translation

Two professional translators were invited to translate the WHO-5 General Well-being Index from Peruvian Spanish into the Collao variant of Quechua spoken in Puno, Peru. The translators speak Peruvian Spanish and have mastered the Collao variant of Quechua spoken in Puno. Each worked on their translation independently. Then they both met with a Peruvian psychologist whose mother tongue is the Collao variant of Quechua spoken in Puno and who has experience in treating anxiety in adults, and a Peruvian psychologist, who is an expert in translating instruments, to put together the proofreading committee. The five of them went through the questionnaire in an in-person meeting and consolidated their work. Upon unanimous approval, a version written in the Collao variant of Quechua spoken in Puno was presented.

#### 2.3.2 Back translation

The consolidated work of the Quechua WHO-5 version was sent to two more translators in the first stage, who performed the back translation from Quechua into Peruvian Spanish. Neither of them had previous knowledge of the study; they presented their translations individually to a member of the research team in an in-person meeting with the participation of a Quechua-speaking psychologist of the variant in question. The translators, the Quechua-speaking psychologist, and the researcher analyzed the similarities between the version translated from Quechua to Peruvian Spanish and the WHO-5 original version in Peruvian Spanish. Once it was confirmed that the items complied with the construct measurement in its back-translated version, they gave their approval to the final version of the Collao variant of Quechua spoken in Puno. Subsequently, two researchers fluent in Quechua and English performed a critical analysis of the items and verified that the Quechua version meant the same as the original English items.

#### 2.3.3 Focus group

A Quechua-speaking psychologist researcher, who had mastered the compilation of qualitative data in focal groups, organized an in-person meeting with seven Quechua speakers (three men and four women) of the Collao variant spoken in Puno. They were all over 18 years old. They had all completed high school; hence, they could read Quechua and were bilingual, speaking Quechua and Peruvian Spanish.

First, they were requested to read the written version of the survey and appropriately answer the two items. After applying the Quechua WHO-5, the psychologist moderator invited the participants to discuss the clarity of the items and their understanding of daily Quechua. The only suggestion was to add the phrase “imatapas ruway munaqlla” to ask about “being in a good mood” in its cultural construction as expressed by Quechua speakers.

#### 2.3.4 Validation of the questionnaire

The validation of the scale included proofreading by experts. Three psychologists (with Master's degrees) participated in this task; two of them had a minimum work experience of two years in the health field, and one of them had experience in university teaching. Their proofreading resulted in a qualitative component for each item, wherein they could make suggestions on the context and relevance of the words in Quechua. It also provided a quantitative component that consisted of the assignment of a certain score for each item. Based on that score, the validity of the content was determined through the relevance, representativeness, and clarity of the items, and the Aiken's V for each item was calculated. Finally, a Collao Quechua version was obtained (Table 2).

**Table 2:** Final translation of the WHO-5 into Puno's Collao Quechua.

No.	Items of the Spanish version	Items in Collao Quechua
1	Me he sentido alegre y de buen ánimo	Kusisqallan karqani, imatapas ruway munaqlla.
2	Me he sentido tranquilo (a) y relajado (a).	Tauqmi kakuni/sintikuni, allin samarisqa hinapuni.
3	Me sentido activo (a) y con energía.	Imapas ruway munaqmi karqani, kallpasapa.
4	Me he levantado sintiéndome bien y descansado(a).	Allinllan hatarini/riqcharini kay tutamantacuna, samarisqa.
5	Mi vida diaria ha tenido cosas interesantes para mí.	Sapa p'unchaymi kawsayñiypi tarini ñuqapaq imapas allintapuni.

Once the scale was translated and validated by the experts, an online form using Google Forms was designed. It included informed consent, a sociodemographic file, and the scale statements. In the first section of the link, the research goal, the fact that participation was anonymous and voluntary, and that the information gathered was for research purposes only were stated. It also stated that only bilingual people, who could at least read Quechua, were allowed to answer the survey.

#### 2.3.5 Data analysis

To validate the content, quantitate data for the assessed items through expert judgment was used. With such data, Aiken's V coefficient was calculated (with significant values ≥ 0,70), taking into account their confidence intervals (CI) at 95%, [35].

Next, descriptive statistical analysis was carried out on the items (mean, standard deviation, skewness, and kurtosis). For skewness and kurtosis, values > ± 2 were considered [24].

Moreover, to determine the validity of the construct, confirmatory factor analysis (CFA) was used through structural equation modeling, using the maximum likelihood estimation method, where it was deemed to consider goodness-of-fit measures, such as the comparative fit index (CFI), Tucker–Lewis index (TLI), goodness of fit index (GFI), and adjusted goodness of fit index (AGFI). The parameters for the root mean square error of approximation (RMSEA) and root mean square error index (RMSE) were also taken into account following the criteria of [25]; who assert that the values of the CFI, TLI, GFI, and AGFI must be greater than 0.90 and RMSEA ≤0.08.

Lastly, the reliability of the construct was determined using the Cronbach's alpha coefficient and its CI [26].

The descriptive analysis was carried out using the statistical program FACTOR Analysis version 10.1, CFA using the AMOS program version 21, and determining reliability using statistical software SPSS version 23.0.

#### 2.3.6 Ethical considerations

This study was approved by the Research Ethics Committee of Universidad Peruana Unión, with approval number 2022-CEUPeU-0038. Additionally, all ethical principles in the Declaration of Helsinki for research in humans were followed.

## 3 Results

Table 3 shows the results of the assessment by three experts, who analyzed the relevance, representativeness, and clarity of the items in the WHO-5 scale. Thus, it was noted that all items received a favorable assessment (V > 0,70). It was specially observed that items 1 and 5 are the most important (V = 1,00; IC 95%: 0,89–1,00), items 1, 5, and 6 are the most representative (V = 1,00; IC 95%: 0,89–1,00), and items 5 and 6 are the clearest (V = 1,00; IC 95%: 0,89–1,00). Therefore, the WHO-5 scale presents evidence of validity based on its content.

**Table 3:** Aiken's V for the assessment of relevance, representativeness, and clarity of the items in the WHO-5 scale.

Items	Relevance (n = 3)				Representativeness (n = 3)				Clarity (n = 3)			
	Mean	SD	V	95% CI	Mean	SD	V	95% CI	Mean	SD	V	95% CI
Item 1	3.00	0.00	1.00	0.89–1.00	3.00	0.00	1.00	0.89–1.00	2.33	0.82	0.78	0.60–0.89
Item 2	2.83	0.41	0.94	0.80–0.99	2.83	0.41	0.94	0.80–0.99	2.17	0.75	0.72	0.54–0.85
Item 3	2.50	0.84	0.83	0.66–0.93	2.50	0.84	0.83	0.66–0.93	2.50	0.84	0.83	0.66–0.93
Item 4	2.33	1.03	0.78	0.60–0.89	2.33	1.03	0.78	0.60–0.89	2.67	0.82	0.89	0.73–0.96
Item 5	3.00	0.00	1.00	0.89–1.00	3.00	0.00	1.00	0.89–1.00	3.00	0.00	1.00	0.89–1.00

SD = Standard deviation.

### 3.1 Preliminary analysis of the items

Table 4 shows the descriptive statistics (mean, standard deviation, skewness, and kurtosis) of the five items of the WHO-5 scale. It is observed that item 4 has the highest average score and highest variability (M = 4.67; DE = 0.95); for skewness and kurtosis scores of the items, they do not exceed the value of ± 2 [24]. Moreover, it is observed that common variance and the corrected correlation coefficient of the item with tall elements is greater than 0.60. Coefficient α the item is removed also presents values greater than 0.80.

**Table 4:** Preliminary analysis of the items in the recovery experiences questionnaire.

Variable	M	SD	A	K	H	r itc	α
Item 1	2.527	0.917	0.026	−0.82	0.599	.722	.866
Item 2	2.581	0.914	0.036	−0.84	0.573	.707	.869
Item 3	2.608	0.946	−0.1	−0.89	0.547	.692	.873
Item 4	2.677	0.952	−0.18	−0.9	0.622	.733	.863
Item 5	2.543	1.011	−0.09	−1.07	0.739	.793	.849

*Note:* M = Mean; SD = Standard deviation; As = Skewness coefficient; K = Kurtosis coefficient; h = Commonality; r itc = Correlation item-corrected test; α = Cronbach alpha.

#### 3.1.1 Confirmatory factor analysis

**Fig. 1:**
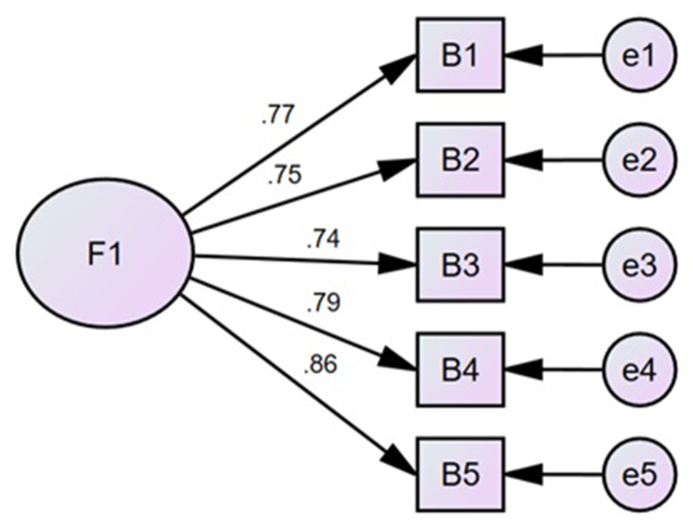
WHO-5 scale model.

To verify the evidence of validity, CFA was performed (Table 5) based on the internal structure of the WHO-5. AGFI confirmed the original one-factor model (χ2 = 10.86, df = 5, p = 0.05; RMR = 0.020; GFI = 0.980; CFI = 0.990; TLI = 0.980; and RMSEA = 0.080). To sum up, the five-item model in a sole latent variable is satisfactory (Fig. 1).

**Table 5:** Adjustment index to the model assessed by the Confirmatory Factor Analysis of the study's instrument.

Model	χ^2^	df	GFI	CFI	TLI	RMSEA	RMR
5 items	10.86	5	.980	.990	980	.080	.020

Lastly, the reliability of the WHO-5 scale was calculated using Cronbach's alpha coefficient, and a value of α = 0.88 (IC 95% = 0.84–0.90) was obtained, which means that the scale is reliable.

## 4 Discussion

[27] highlights the growing interest in the study of subjective well-being as an indicator of people's physical and mental health. This raises the relevance of having valid and reliable instruments for its measurement, which can be used in various cultural and geographical environments, and hence the translation of such instruments into native languages is required. In this regard, the objective of this study was to translate and validate the WHO-5 Well-Being Index into Quechua, in its Collao variant, a language spoken in the indigenous communities of southern Peru.

After obtaining favorable evidence of the relevance, representativeness, and clarity of the items translated into Collao Quechua, through the criteria of expert judgment, the analysis of the psychometric properties showed acceptable indicators. The validity evidence based on the internal structure were obtained through the CFA, the results of which show appropriate fit indices for the unidimensional model, that is, the WHO-5 items translated into Collao Quechua made up appropriate indicators to measure subjective well-being as the only latent characteristic. This confirms what previous studies have found at national [19] and international [20,21,[28], [29], [30]] level that it is consistent with the original version designed by the World Health Organization [31] as a single dimension instrument considering five indicators to measure well-being in primary assistance. Additionally, taking into account the study where [19] suggest the correlation of error in items 1 and 4, whereas the research of [29] suggests the correlation of error in items 3 and 4, the modification indexes were analyzed, and it was found that it was not necessary to model the correlation among the residuals of the items translated into Collao Quechua. This proves that the correlations among residuals of different items can vary depending on the characteristics of the sample groups and that cultural and linguistic factors are relevant. Furthermore [32], points out that similarity in item phrasing is a source that can account for the covariance between items. Based on the findings and their consistency with the background results, it is considered that the unidimensional structure of the WHO-5 translated into Collao Quechua has reasonable empirical support.

As for measurement accuracy, the research showed that the WHO-5 translated into Collao Quechua presents good reliability from an internal consistency perspective and considering the value of Cronbach's alpha coefficient (α = 0,88), which has a value similar to that obtained for the translation into Singhala [29] and the Spanish version [28,30]. Therefore, it is understood that the WHO-5 translated into Collao Quechua is a reliable instrument.

This research had certain limitations that should be reported. First, the small size of the sample and the nonprobabilistic nature of participant selection limit the generalization of the results obtained because all participants came from the Puno region. Therefore, we suggest assessing the psychometric properties of the WHO-5 translated into Collao Quechua in different geographical contexts where this language is spoken, such as the communities in the southern Peru, specifically Apurímac, Cusco, Arequipa, and Moquegua. Second, only the validity evidence based on the content and internal structure was tested. Therefore, it is recommended that future studies complement the findings obtained with other instruments or external criteria, such as medical diagnosis, to collect more evidence on validity based on the relationship with other variables. This would provide more information on the variables that have proved to have a strong association with the WHO-5 measure, such as depression and anxiety [18]. Moreover, factor invariance has not been analyzed based on some relevant sociodemographic characteristics such as sex, age group, or occupation. The reliability of the WHO-5 translated into Collao Quechua has not been studied using the test–retest technique, so a conclusion on the mean stability cannot be reached. We suggest adding both aspects to the future pending research agenda.

In spite of the abovementioned limitations, the results of this study are relevant. The practical implications of the research are mainly about supplying an instrument translated in Collao Quechua with evidence of validity and reliability to measure the subjective well-being among the population in the Peruvian South who speak this language. In such way that the results allow primary care personnel to have a brief, simple, and useful measurement tool for the evaluation of aspects linked to the subjective well-being of ethnic minorities, which could provide an indicator for the timely detection of possible psychological problems, leading to rapid intervention and treatment, as previous studies have highlighted the usefulness of the WHO-5 as a sensitive and specific screening tool for depression [18]. Therefore, the importance of measurement instruments that are culturally adapted for indigenous and ethnic minorities and that contribute to their well-being is highlighted.

To conclude, the WHO-5 translated into Collao Quechua has acceptable psychometric properties, and it can be used as a welfare measurement tool for the indigenous peoples of south Peru that speak this language.

## Author contribution statement

Renzo Felipe Carranza Esteban; Oscar Mamani-Benito; Julio Cjuno; Madona TitoBetancur; Susana K. Lingán-Huamán; Dennis Arias-Chávez: Conceived and designed the experiments; Performed the experiments; Analyzed and interpreted the data; Contributed reagents, materials, analysis tools or data; Wrote the paper.

## Data availability statement

The authors are unable or have chosen not to specify which data has been used.

## Declaration of competing interest

The authors declare that they have no known competing financial interests or personal relationships that could have appeared to influence the work reported in this paper.
